# A Conduction‐Nucleation‐Competition‐Guided 3D Adaptive Gradient Buffer for Dendrite‐Free All‐Solid‐State Lithium Metal Batteries

**DOI:** 10.1002/advs.77775

**Published:** 2026-09-27

**Authors:** Yaru Shi, Libin Hu, Qiuhong Li, Zheng Zhang, Shoushuang Huang, Bing Zhao, Yi Xu, Zhangjun Hu, Yong Jiang, Jiujun Zhang

**Affiliations:** ^1^ School of Environmental and Chemical Engineering Shanghai University Shanghai China; ^2^ Division of Molecular Surface Physics & Nanoscience Department of Physics Chemistry and Biology Linköping University Linköping Sweden; ^3^ Institute For Sustainable Energy/College of Sciences Shanghai University Shanghai China; ^4^ Institute For New Energy Materials and Engineering College of Materials Science and Engineering Fuzhou University Fuzhou China

**Keywords:** adaptive gradient buffer layer, conduction‐nucleation competition mechanism, dynamic self‐optimization, solid‐state lithium metal batteries

## Abstract

The existing designs of buffer for all‐solid‐state Li‐metal batteries (ASSLMBs) are mostly limited to static optimization of single functions and lack systematic regulation of the dynamic competition among ionic conduction, electron distribution, and interfacial lithiophilicity. Herein, a conduction‐nucleation competition mechanism is proposed firstly, clarifying that lithium deposition is governed by the synergy of these three factors. Guided by this principle, a self‐adaptive gradient buffer layer composed of soft carbon and Mg_3_N_2_ (SCMN) is designed. After lithiation, the buffer forms a continuous lithiophobic/lithiophilic gradient and subsequently undergoes *in‐situ* conversion into highly ion‐conductive Li_3_N and highly electron‐conductive/ lithiophilic Li‐C/Li‐Mg alloys during electrochemical activation, enabling dynamic interface self‐optimization. The gradient structure creates a fast ion–electron dual conduction network, guiding bottom‐top lithium deposition within the buffer layer without volume expansion. Consequently, the symmetric cells achieve stable cycling over 1600 h at 1.5 mA cm^−^^2^ and remain stable even under low stack pressure (15 MPa). The LiCoO_2_‐based full cells deliver ultra‐stable cycle with 98.5% capacity retention after 200 cycles at 0.1 C. Additionally, the full cells paired with high‐voltage LiNi_0.8_Co_0.1_Mn_0.1_O_2_ cathodes exhibit exceptional cycling stability at high loading (4.45 mg cm^−2^). This work demonstrates a mechanism‐driven design paradigm toward high‐energy, long‐life ASSLMBs.

## Graphical abstract

A self‐adaptive gradient SCMN buffer layer is designed based on a conduction‐nucleation competition mechanism. The design dynamically optimizes ion/electron conduction and lithiophilicity, guiding dendrite‐free lithium deposition. Consequently, symmetric cells deliver exceeding 1600h at 1.5 mA cm^−2^, and the LiCoO_2_‐based full cells exhibit 98.5% capacity retention after 200cycles.

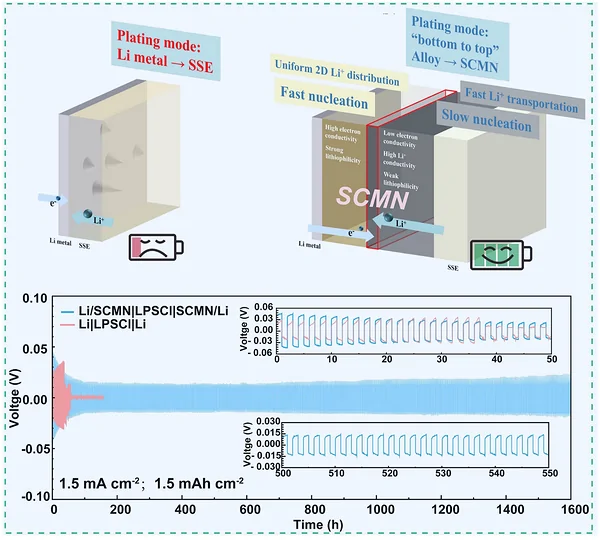

## 1 Introduction

All‐solid‐state lithium metal batteries (ASSLMBs) employing non‐flammable solid electrolytes (SEs) and lithium metal anodes with exceptionally high theoretical capacity (3860 mAh g^−1^) and low redox potential (‐3.04 V vs. SHE) demonstrate significant potential for achieving enhanced energy density and improved safety [1, 2, 3]. Among various SEs, sulfide‐based systems are particularly promising due to the high room‐temperature ionic conductivity (1∼25 mS cm^−1^) and favorable mechanical properties [4]. The sulfide electrolytes can be readily cold‐pressed into dense, low‐porosity film, effectively reducing interfacial resistance [5, 6, 7, 8].

**FIGURE 1:**
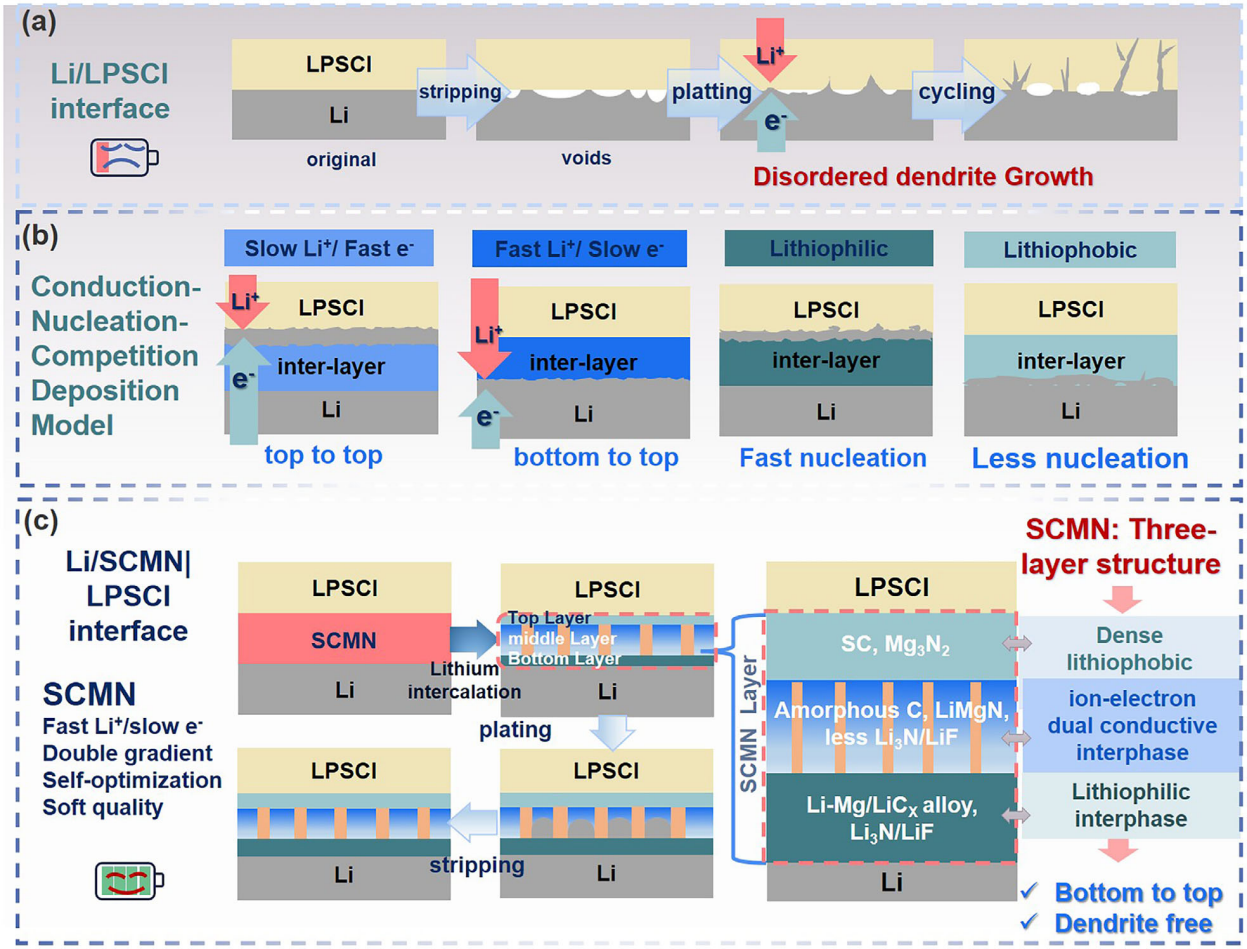
Schematic illustration of Li deposition for (a) LPSCl/Li interface; (b) Conduction‐nucleation‐competition deposition model; (c) Li/SCMN|LPSCl interface. The SCMN buffer layer transforms into SC/Mg_3_N_2_ top layer, LiMgN/Li_3_N‐rich middle layer, and LiC_6_/Li‐Mg alloys and Li_3_N‐rich bottom layer after activation, which exhibit dense lithiophobic (top), fast ion–electron dual‐conductive (middle) and lithiophilic (bottom) gradient properties respectively, thus regulating a bottom‐up Li deposition within the buffer layer.

However, the narrow electrochemical window of sulfide SEs renders them prone to parasitic interfacial side reactions when paired with highly reductive lithium metal anodes [9, 10]. Simultaneously, the uncontrolled growth of lithium dendrites presents another critical bottleneck to the commercialization of sulfide‐based ASSLMBs [11, 12]. In the systems, lithium deposition tends to concentrate at the Li/SEs interface, further elevating the risk of charge accumulation and severely limiting the rate capability of the ASSLMBs [13, 14]. The interfacial lithium deposition behavior stems from the low diffusion coefficient of Li^+^ within the lithium metal anode, which slows Li^+^ transport into the bulk and promotes premature reduction upon contact with electrons at the interface [15, 16]. Especially under high current densities, the accelerated rate of Li^+^ reduction to metal lithium substantially increases the likelihood of dendrite nucleation and growth, ultimately leading to a sharp decline in battery performance or even failure (Figure 1a).

To address these challenges, prevailing strategies often focus on modifying the solid electrolyte interphases (SEI) [17, 18, 19] or alloying the anodes [20, 21]. However, both approaches are fundamentally constrained by inadequate ion/electron transport matching and a lack of additional deposition capacity, rendering them insufficient to meet the practical requirements of high current density and high areal capacity. To overcome the challenge, constructing artificial buffer layer [22, 23, 24] between the lithium metal anode and the SEs has emerged as a more promising solution, which not only provides physical isolation to suppress interfacial side reactions but also offers substantial design flexibility [25]. These buffer layers include carbon, metal particles, carbon‐metal composites, and other inorganic materials. Among them, low‐cost carbon materials [26, 27] are considered excellent buffer layer matrixes due to their low density, good ion conductivity, and lithium insertion behavior. Lee et al. [28] developed a Ag‐C composite buffer layer and demonstrated that the thin Ag‐C buffer layer effectively regulates Li deposition. In addition, Avvaru et al. [29] demonstrated that lithiophilic alloys should be positioned away from the SE interface to prevent dendrites by strategically arranging Sn and C in a hierarchical structure.

Guiding lithium deposition to preferential locations is critically important for ASSLMBs. However, it is still challenging to simultaneously create lithiophobic regions within a buffer layer to suppress harmful nucleation at the interface, as well as create lithiophilic regions to guide uniform deposition of lithium, while ensure efficient and directional transport of both lithium ions and electrons toward these pre‐defined sites. The fundamental reason is that the nucleation sites and deposition morphology of lithium are not determined by a single factor, but rather by the dynamic competition and synergy of ion transport pathways, electron distribution networks, and interface lithium affinity (Figure 1b). At present, there is a lack of a collaborative mechanism and universal design principles that can simultaneously and dynamically regulate the above three factors. The existing buffer layer designs is unable to fundamentally solve the interwoven problems of ion/electron loss distribution, insufficient deposition space supply, and continuous dynamic failure of interfaces in cycling under high surface capacity conditions. At present, a coherent mechanism and general design principles capable of simultaneously and dynamically coordinating these three factors are still lacking. Consequently, the buffer‐layer designs fail to fundamentally resolve the intertwined challenges of ion/electron flux mismatch, inadequate deposition space, and continuous interfacial degradation under high‐areal‐capacity cycling.

Herein, an adaptive composite gradient buffer layer using soft carbon (SC) and Mg_3_N_2_ as precursors was prepared based on conduction‐nucleation competition mechanism. After preprocessing, the buffer layer spontaneously forms a continuous lithiophobic/lithiophilic functional gradient, and subsequently converts into high ion conductor Li_3_N and high electron conductivity, strongly lithiophilic Li‐C/Li‐Mg alloys via *in‐situ* lithium intercalation reactions, thereby achieving dynamic self‐optimization of interface properties and continuous enhancement of functionality. Density functional theory (DFT) calculations confirm the high lithiophilicity of the resulting alloy phases and the role of Li_3_N as a fast ion conductor. Benefiting from the lithiophobic‐to‐lithiophilic gradient, the formation of a fast Li_3_N/Li‐Mg alloys ion‐electron dual‐continuous network, and the confinement effect of the SC framework, lithium deposition is guided to proceed uniformly in a bottom‐up, volume‐neutral manner within the buffer layer (Figure 1c). As a result, the Li||Li symmetric cell exhibits stable long‐term cycling over 1600 h at 1.5 mA cm^−2^ and remain stable cycle even under low stack pressure. Furthermore, both the LCO and NCM811 full cells achieve exceptional cycling stability over 200 cycles at high areal loading, demonstrating the practical effectiveness of this interface‐engineering strategy for high‐performance ASSLMBs.

## 2 Results and Discussion

### 2.1 The Formation and Characteristics of the Self‐Delaminating Structure of the SCMN Buffer Layer

**FIGURE 2:**
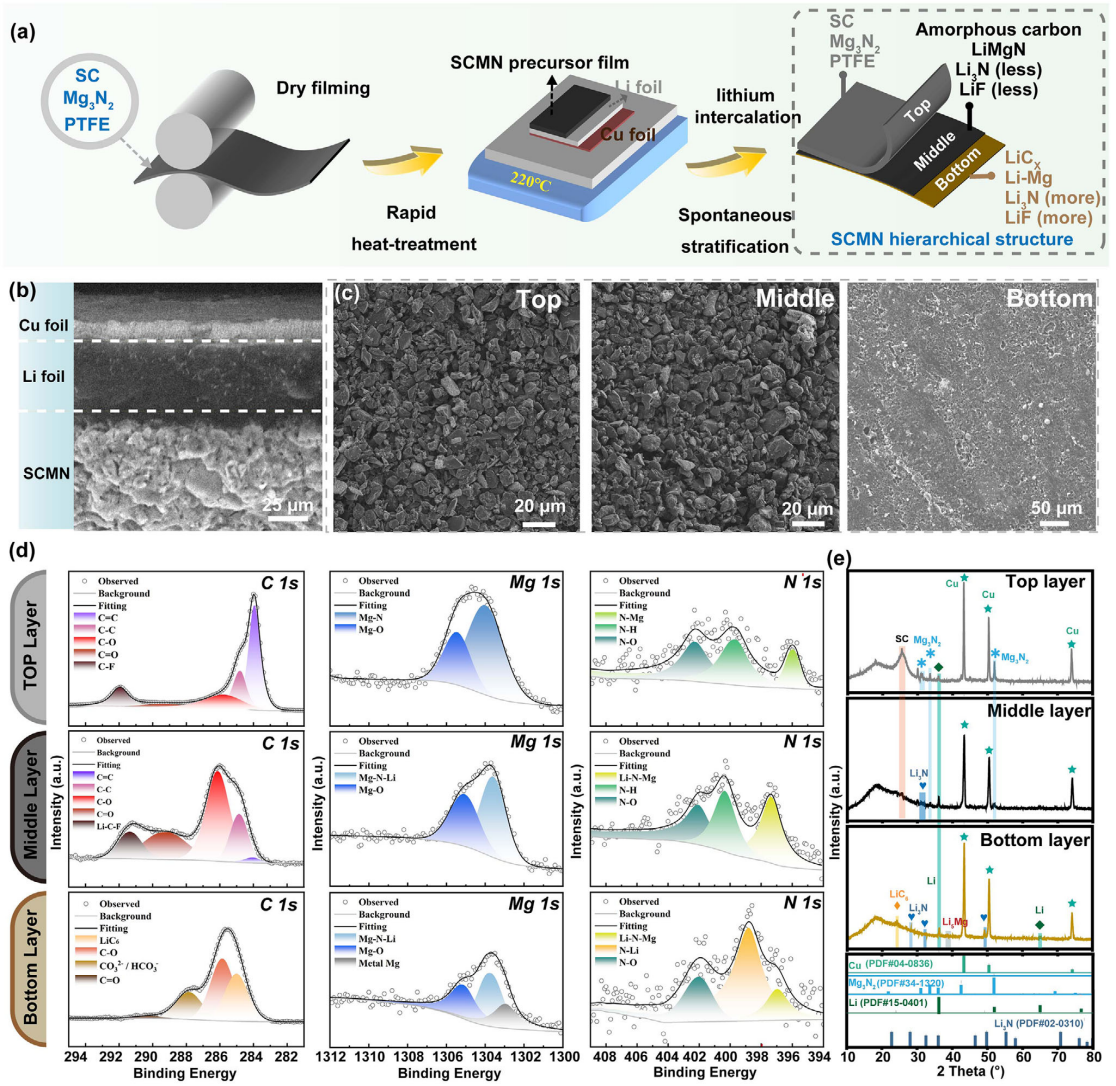
(a) Schematic diagram of the formation process of electron‐ion 3D gradient buffer layer; (b) Cross‐view SEM image of the Li/SCMN anode; (c) Top‐view SEM images of top‐middle‐bottom layers of the SCMN buffer layer; (d) XPS spectra, and (e) XRD patterns of the top layer, middle layer and bottom layer in the buffer layer.

The SC/Mg_3_N_2_/PTFE film, prepared by dry‐film formation process and denoted as SCMN, was sequentially laminated omto lithium‐coated copper foil and then subjected to rapid heat treatment at 220 °C for 30 s using a hot press, yielding an integrated composite anode (Figure 2a). After treatment, the original SCMN layer spontaneously delaminated into a three‐tier structure. The top layer retains a film‐like morphology with a dark gray appearance and could be peeled off mechanically. Beneath this layer, a black powdery residue layer was exposed, suggesting loss of structural integrity in this region. Further removal of this powdery layer reveals a yellowish‐brown, dense and hard surface, distinctly different in morphology from the pristine lithium metal. These three layers are labeled as the “Top layer,” “Middle layer,” and “Bottom layer,” respectively (the surface optical images are shown in Figure S1).

To systematically investigate the stratified architecture, scanning electron microscopy (SEM) was employed to examine both surface and cross‐sectional morphologies. The cross‐sectional SEM image of the integrated anode coupled with the Li_6_PS_5_Cl (LPSCl) electrolyte pellet clearly reveals a multi‐layer stack comprising the copper foil (∼10 µm), lithium foil (∼45 µm), SCMN buffer layer (∼50 µm), and the electrolyte (Figure S2a). The peeling process further confirms the layered structure (Figure S2b), where the folded top film is observed in the upper‐right region and the exposed intermediate layer appears in the lower‐left region. Both the top layer and the middle layer exhibit similar granular morphology consisting of particles 5–10 µm in size. In contrast, after complete removal of the intermediate residue, the underlying layer displays a relatively smooth and dense surface (Figure 2c). These observations provide direct evidence for the formation of a distinct three‐layer architecture with pronounced structural gradients. Such a stratified structure originates from the in‐situ thermochemical reaction between the SCMN buffer layer and molten lithium during rapid thermal treatment, which drives spontaneous phase and morphology evolution across the electrode thickness.

To further clarify the precise chemical composition of each layer, X‐ray photoelectron spectroscopy (XPS), Raman spectroscopy, and X‐ray diffraction (XRD) analyses were systematically performed. As displayed in Figure 2d, the top layer shows characteristic peaks of C = C at 284.0 and 284.8 eV (graphitic) and C–C (amorphous carbon/PTFE), along with weak C–O (∼ 286.0 eV), C = O (∼288.0 eV) signals and a C–F peak at ∼292.0 eV from PTFE. In the middle layer, the intensity of the C = C decreases markedly while that of C–C increases, reflecting a reduction in graphitic order and the dominance of disordered carbon structures [30]. Concurrently, the C–F peak shifts from 292.0 to ∼291.0 eV, confirming the formation of a Li–C–F intermediate due to the reaction between molten lithium and PTFE. In the bottom layer, the emergence of a characteristic LiC_6_ peak at 285.2 eV, along with the disappearance of C–C/C = C signals, indicates the transformation into a lithium–carbon alloy [31]. The high reactivity of this alloy leads to rapid surface oxidation and hydrolysis upon air exposure, as evidenced by pronounced signals from oxygen‐containing functional groups (C–O, CO_3_^2^^−^/HCO_3_^−^, C = O). F 1s spectra show a transition from only C–F (top layer) to Li–C–F and LiF (intermediate layer), and finally to dominant LiF (bottom layer), reflecting progressive PTFE reduction by molten Li (Figure S3) [32]. Notably, although the pristine PTFE component is consumed during the thermal reaction, the in‐situ generated LiF serves as an ideal active component, endowing the underlying layer with high chemical stability and excellent electrical insulation, a highly beneficial functional product for lithium metal anodes. Besides, PTFE serves dual functions in this system. In the top layer, it retains structural role as a binder, maintaining film integrity. In the bottom layer, it is reductively defluorinated by molten Li to form LiF, which serves as a stable SEI component. This spatial separation of functions is enabled by the Li concentration gradient across the SCMN layer, confining PTFE degradation to the bottom region without compromising overall structural integrity. Mg 1s spectra show that the top layer presents peaks at 1303.5 eV (Mg–N) and 1304.5 eV (Mg–O), followed by a ∼0.4 eV shift in Mg–N toward lower binding energy in middle/bottom layers due to Li substitution forming Li–N–Mg [33]. Additionally, Metallic Mg^0^ appears at 1303.0 eV in the bottom layer, confirming the presence of reduced metallic magnesium adjacent to the lithium metal. Li 1s and N 1s spectra further support this evolution (Figure 2d and Figure S3): Li shifts from electrolyte/oxide species (top) to Li–N–Mg/Li–C–F intermediates (middle) and finally to LiC_6_, LiF, and Li_3_N (bottom). N species transition from Mg–N (top) to Li–N–Mg (middle) and predominantly Li_3_N (bottom), with N–O signals present throughout due to Mg_3_N_2_ hydrolysis [34]. Adsorbed N species are only detected in the carbon‐containing top and intermediate layers.

The XRD patterns reveal distinct crystallographic differences among the layers (Figure 2e). In the top layer, the hump centered at 2θ ≈ 18° originates from the Kapton tape [35], and the relatively narrower hump at 2θ ≈ 25° corresponds to the characteristic diffraction of SC within the SCMN layer. The sharp peaks are assigned to Cu foil (JCPDS No. 04–0836), Mg_3_N_2_ (JCPDS No. 34–1320) [33]. The absence of any new diffraction peaks indicates that no detectable chemical reactions occurred in the top film during rapid thermal treatment, thus preserving the phase composition of the precursor mixture. The middle layer, obtained after removal of the top film, retains the primary inorganic phases (Mg_3_N_2_, Cu foil), but shows a broader and weaker SC diffraction signal, suggesting declined carbon crystallinity, increased amorphous carbon, and incomplete Mg_3_N_2_ reaction. The bottom layer adjacent to lithium anode shows distinct phase transformation. Most original diffraction signals vanish, replaced by new phases. The SC peak at 25–26° is replaced by one at 24.0° from LiC_6_ (JCPDS No. 25–0523 [30], indicating full lithium intercalation. Peaks around 38.5° emerge, assigned to a lithiated Li_5_Mg alloy (Figure S4), and Li_3_N peaks appear at 23.2° and 31.5° [36, 37], which confirms alloying reaction occurred in the bottom layer during rapid thermal treatment.

Raman spectroscopy (Figures S5 and S6) further elucidates the phase evolution within the stratified structure. The top layer maintains pristine SC and Mg_3_N_2_. The middle layer exhibits attenuated Mg_3_N_2_ peak at 379 cm^−1^ and an elevated D‐to‐G band intensity ratio (I_D_/I_G_) of 1.32 [38], indicating partial Mg_3_N_2_ consumption and increased carbon defects, consistent with XRD results. The bottom layer undergoes dramatic structural changes. The graphitic G band completely vanishes, replaced by a broad D‐band feature. The peaks observed at ∼205, 1570 and 1605 cm^−1^ are assigned to the highly lithiated compound LiC_6_ [39], while the broader signal at higher wavenumbers corresponds to a series of partially lithiated LiC*_x_* alloys. Additionally, distinct low‐wavenumber peaks attributed to Li_3_N are clearly detected, confirming the concentrated formation of lithium nitride within the alloy layer.

In short, the rapid thermal treatment induces a spatially gradient reaction rather than uniform modification. The top layer near the electrolyte retains the intrinsic phase of the precursor (SC, Mg_3_N_2_ etc.), serving as an ion‐sieving and physical isolation layer; The middle layer undergoes amorphization of the carbon framework and partial lithiation of Mg_3_N_2_, with the main composition of LiMgN and Li_3_N, which act as rapid ion transport channels; while the bottom layer in contact with lithium metal is completely transformed into lithiophilic alloy composite phases (Li‐C, Li‐Mg and Li_3_N etc.), which is clearly an ideal host for lithium deposition. The layer‐by‐layer evolution from “inert” to “active” constitutes a built‐in, adaptive functional gradient system.

**FIGURE 3:**
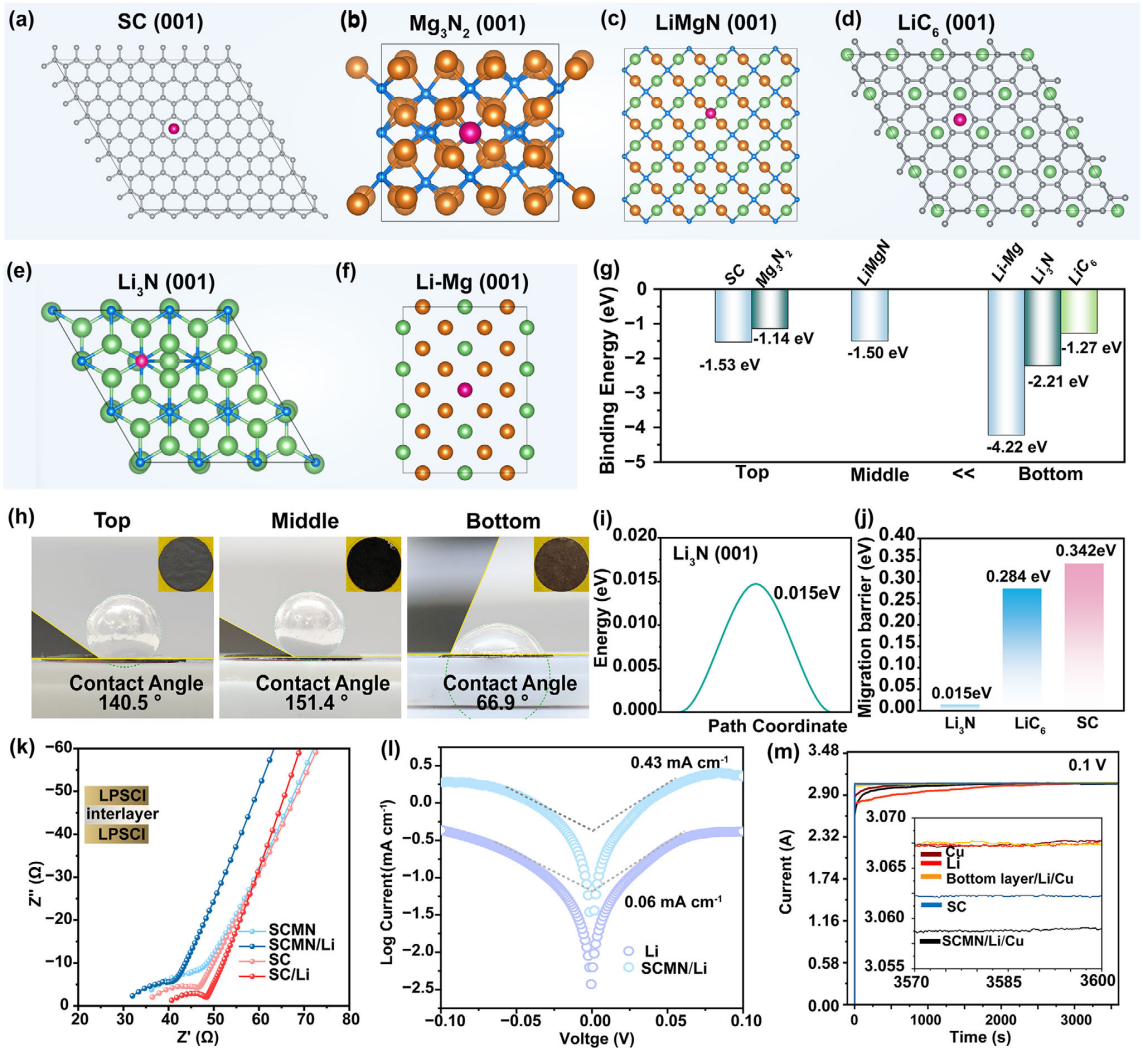
(a‐f) Adsorption structure models of lithium atoms on SC (001), Mg_3_N_2_ (001), LiMgN (001), LiC_6_ (001), Li_3_N (001), LiMg_2_ (010) surfaces and (g) Comparison of binding energy; (h) Contact angle test of molten lithium on the surface of each layer; (i) Lithium‐ion migration barriers of Li_3_N and (j) corresponding comparison with SC and LiC_6_; (k) Electrochemical impedance of LPSCl|interlayer|LPSCl electronic blocking cells; (l) Tafel curves; (m) DC polarization curve.

To further understand the influence of components on the functions of each layer, DFT calculations [40, 41, 42] were conducted. Figure 3a–g reveal distinct lithiophilicity evolution during phase transitions: the binding energy for Li^+^ shifts from ‐1.53 eV (SC) to ‐1.27 eV (LiC_6_), indicating enhanced lithiophilicity upon carbon lithiation. Conversely, Mg_3_N_2_ derivatives exhibit progressively enhanced lithiophilicity, with binding energies of ‐1.50 eV (LiMgN), ‐2.21 eV (Li_3_N), and ‐4.22 eV (LiMg_2_). Metallic Mg shows extreme lithiophilicity (‐5.67 eV *vs*. Li: ‐0.61 eV) (Figure S7). Analysis of the stratified structure confirms weaker lithiophilicity in the top/middle layer (carbon‐containing species, Mg_3_N_2_, LiMgN) vs. strong lithiophilicity in the bottom layer (Li*_x_* Mg*_y_*, Li_3_N) [43]. Molten lithium contact angle measurements (Figure 3h) also corroborate this gradient: 140.5° (top layer), 151.4° (middle layer), and 66.9° (bottom alloy layer). The in‐situ lithiated/lithiophilic gradient structure forms a nucleation barrier difference on the upper and lower surfaces of the buffer layer. Lithium‐ion intercalation into soft carbon (ΔG = –2.29 eV) is energetically more favorable than adsorption on the graphite surface, confirming that lithium preferentially inserts into the carbon framework rather than nucleating on the surface. The process not only suppresses lithium deposition at the interface but also reduces the apparent lithiophilicity of the interlayer due to the formation of LiC*_x_* alloys (Figure S8).

The migration energy barrier for lithium ions in Li_3_N is extremely low, merely 0.015 eV (Figure 3i), a value significantly lower than the typical migration barriers in graphite intercalation compounds (such as LiC_6_, 0.284 eV) and SC (0.342 eV) (Figure 3j and Figure S9), confirming that Li_3_N is a fast ion conductor in the system [44]. To electrochemically evaluate the ionic conductivity of the interlayer, symmetric cells were assembled by sandwiching the SCMN buffer between two LPSCl SE pellets, with stainless steel rods serving as ion‐blocking electrodes for electrochemical impedance spectroscopy (EIS) measurement (Figure 3k). The impedance of the SCMN/Li after lithium injection is significantly lower than that of the pristine SCMN layer. The impedance reduction is attributed to the formation of fast ion‐conducting phases such as Li_3_N and LiC_6_ during the molten lithium reaction, which substantially enhance ion‐transport pathways across the interlayer. In contrast, a control sample consisting solely of SC subjected to the same process exhibits markedly higher impedance, further underscoring the indispensable role of the in‐situ generated Li_3_N in optimizing the overall ionic conductivity of the SCMN buffer layer. The Tafel curves of the symmetric cells indicate that compared to the bare lithium anode, the SCMN@Li anode exhibits a higher exchange current density (0.43 mA cm^−2^, Figure 3l). This is because the three‐dimensional ionic network enhances the rate of lithium‐ion transport, and the lithium‐friendly underlying layer increases the deposition sites for lithium metal.

DC polarization time‐current curves indicate that SCMN@Li exhibits the lowest polarization current (Figure 3m and Figure S10). In contrast, the polarization current of the bottom alloy layer increases markedly, approaching values comparable to those of Li/Cu and pure Cu, demonstrating that the bottom layer possesses high electronic conductivity similar to that of a pure metal. Further measurements of the electronic conductivity of the pure SC with equivalent thickness confirm that its conductivity is significantly lower than that of the bottom alloy layer. The results indicate that the bottom alloy layer provides high electronic conductivity and strong lithiophilicity, which thermodynamically favors lithium nucleation. Concurrently, the embedded Li_3_N phase offers ultra‐low ionic migration barriers, while the overall composite ensures rapid bulk ion transport, kinetically enabling sufficient Li^+^ supply to the nucleation sites. Therefore, lithium deposition is neither limited by the high‐resistance Li/SCMN interface nor thermodynamically preferential to the upper layer with weaker lithiophilicity. Instead, it is actively guided to the bottom alloy layer, where rapid ion conduction, efficient electron supply, and high nucleation affinity are concentrated. This synergistic effect of multiple properties successfully guides the deposition of active lithium from the Li/SE interface to the interior of the functionalized buffer layer, reflecting a rational interface design based on the competitive regulation of ion diffusion, electron transport and lithium affinity at the interface.

### 2.2 The Lithium Deposition Regulation and Self‐Optimization Behaviors of SCMN‐Li Buffer Layer

To further evaluate the regulate effect of the SCMN buffer layer on lithium deposition behavior, half‐cells and symmetric cells were assembled. Half‐cell tests reveal the unique two‐step “intercalation‐deposition” mechanism of the SCMN layer. Figure S11 presents the capacity‐voltage curves of the half‐cells with/without SCMN layer, both deposits at a current density of 1.5 mA cm^−2^ until short‐circuit. The SCMN integrated half‐cell exhibits exceptional lithium deposition tolerance, reaching 5.7 mAh cm^−2^ at short‐circuit, far exceeding the 1.36 mAh cm^−2^ of bare Li metal. The improvement stems from the elastic, porous SCMN buffer layer, which accommodates lithium deposition while enabling additional Li storage via carbon intercalation. Notably, the SCMN half‐cell shows a pronounced polarization rise before short‐circuit, caused by void formation in the working Li metal electrode during stripping, which degrades interfacial contact and increases impedance. Short‐circuiting is thus primarily driven by the working electrode, not failure of the SCMN reference anode. In the magnified view, Li metal deposition shows negligible initial overpotential, whereas SCMN deposition displays a stepped overpotential characteristic of carbon material lithiation. Further morphological analysis was conducted on SEs after disassembling the half‐cells. Benefiting from the physical isolation effect of the SCMN layer, the surface of the SE remains smooth and flat after lithium deposition (Figure S12a). In contrast, the surface of the unprotected SE is severely eroded by lithium dendrites (Figure S12b). Dynamic SEM observations of the SCMN surface under different deposition capacities reveals its structural evolution (Figure S13): as the deposition capacity increas from 0 to 3 mAh cm^−^^2^, the particles in the membrane swells and their boundaries gradually blurs, directly visualizing the in‐situ transformation process of the SC into lithium‐carbon alloy (LiC_6_).

**FIGURE 4:**
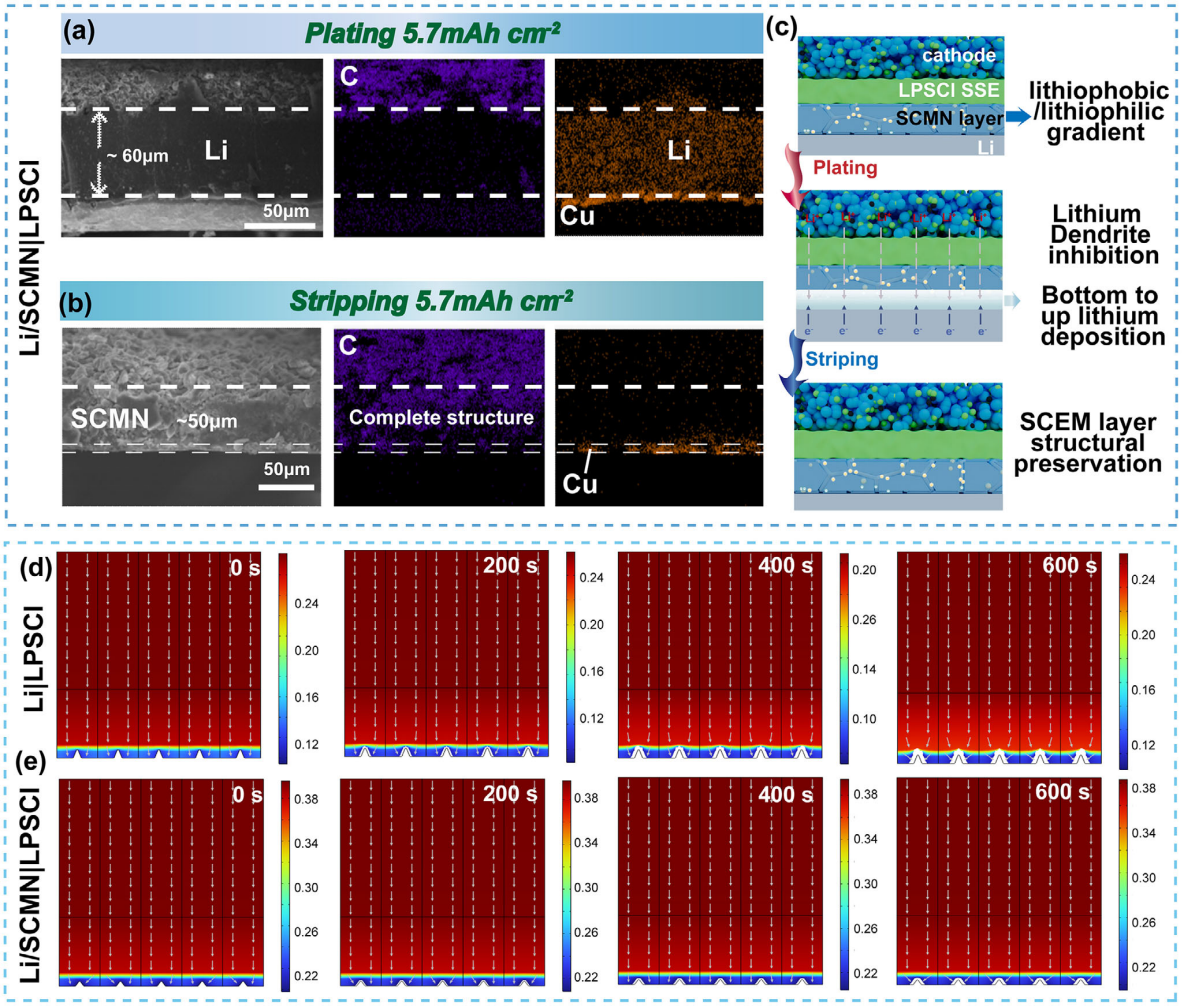
(a) Cross‐section SEM of the Li/SCMN anode after Li plating, and the corresponding EDS mapping; (b) Cross‐section of the Li/SCMN anode stripped 5.7mAh cm^−2^ with EDS mapping; (c) Schematic diagram of lithium plating/striping process in the LPSCl/SCMN‐Li interface; COMSOL simulations of the lithium deposition process of the (d) Li and (e) Li/SCMN anode surface.

To verify the deposition sites of lithium ions in Li/SCMN|LPSCl interface, the cross‐section SEM of the symmetric cells undergoing extreme depositing‐stripping cycles were observed (Figure 4a,b). It is worth noting that after deposition at 5.7 mAh cm^−2^, the total thickness of the electrode only increases by ∼10 µm. According to Faraday's law, if an equal amount of lithium is deposited on the surface in pure metallic form, its theoretical thickness should increase by ∼27.6 µm [45]. The minor thickness variation clearly indicates that the deposited lithium is primarily stored within the alloy phases of the SCMN buffer layer (LiC_6_ and Li‐Mg alloy). These alloy phases possess substantially higher density than pure lithium, leading to markedly lower volume expansion when accommodating the same quantity of lithium. This near‐zero volume expansion on the anode side is a key advantage of our design, as it effectively mitigates the mechanical stress accumulation that commonly plagues conventional Li metal anodes. From the perspective of macroscopic dimensional change, the finding directly validates the success of the conduction‐nucleation competition mechanism, demonstrating that lithium is effectively guided and stored within the 3D host structure of the buffer layer, rather than accumulating as a 2D layer at the interface (Figure 4c).

To gain a deeper understanding of the intrinsic mechanism of lithium deposition behavior, CMOSOL phase field simulations were conducted to investigate the regulatory effect of SCMN gradient buffer layer on lithium deposition morphology and current distribution [46]. At the SCMN/Li interface, lithium deposition exhibits a uniform planar growth mode, and the current density is evenly distributed at the electrode interface without obvious local concentration, indicating effective suppression of dendrite growth (Figure 4e). In sharp contrast, at the LPSCl/Li interface without a buffer layer, simulation results show a typical dendritic deposition morphology, accompanied by highly localized current density distribution. The uneven charge distribution leads to significant dendritic growth (Figure 4d). The above results collectively demonstrate that the SCMN buffer layer can achieve rational regulation of lithium deposition behavior from both thermodynamic and kinetic dimensions.

**FIGURE 5:**
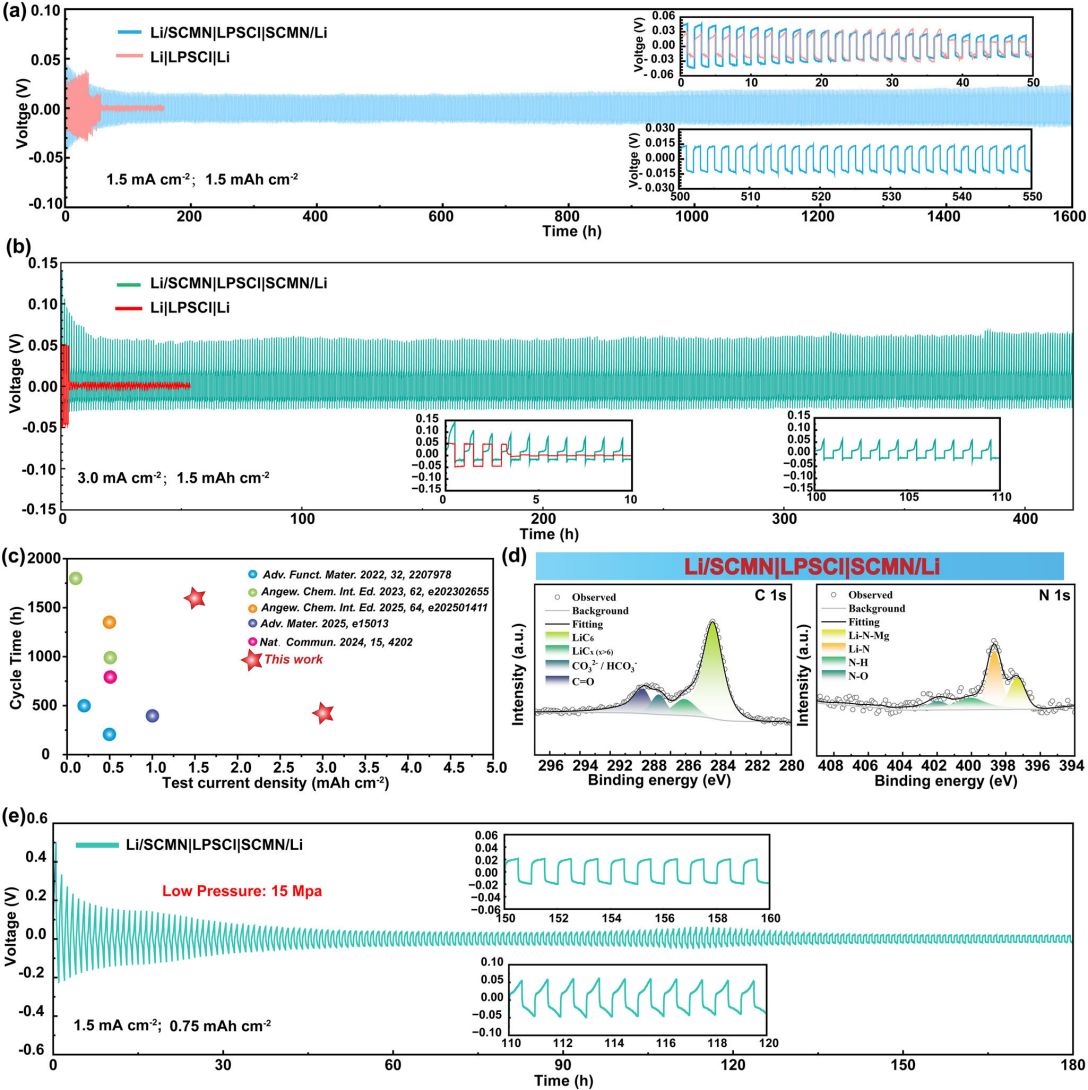
Cycling performance of lithium symmetric cells with/without SCMN layer at (a) 1.5 mA cm^−2^, 1.5 mAh cm^−2^ and (b) 3.0 mA cm^−2^, 1.5 mAh cm^−2^; (c) Performance comparison of sulfide electrolytes reported in previous works; (d) C 1s and N 1s XPS spectra of the SCMN surface after cycling; (e) Cycling performance of lithium symmetric cells with SCMN buffer layer under low stacking pressure of 15 MPa.

The symmetric cells with and without SCMN layer were performed for galvanostatic cycling. As shown in Figure 5a, under 1.5 mA cm^−2^, 1.5 mAh cm^−2^, the Li symmetric cell with SCMN layer initially exhibits higher polarization than the Li|LPSCl|Li symmetric cell, attributable to the additional interfacial impedance of the SCMN layer. Nevertheless, the polarization steadily decreases during cycling, and the Li symmetric cell with SCMN layer eventually achieves stable cycling over 1600 h, demonstrating excellent long‐term stability. In contrast, the Li|LPSCl|Li symmetric cell displays continuously increasing polarization from the outset, indicating severe interfacial side reactions forming resistive interphases [47, 48], compounded by dendrite growth that penetrates the electrolyte after only 37 cycles. At the ultra‐high current density of 3.0 mA cm^−2^, 1.5 mAh cm^−2^ (Figure 5b), the Li |LPSCl| Li cell experiences an internal short circuit after merely a few cycles. Remarkably, the SCMN symmetric cell, after an initial ∼20 cycles of polarization reduction (activation), stabilizes and cycles for over 400 h, surpassing most reported sulfide electrolytes in the literature (Figure 5c). Voltage fluctuations are limited to the polarization overshoot component, and the baseline potential exhibits abnormal stability, which confirms that the SCMN interlayer persistently protects the solid electrolyte interface under high current/high capacity enabling long‐term stability for lithium metal anodes in all‐solid‐state batteries.

To reveal the underlying mechanism of the decrease in polarization of the SCMN cells during lithium plating/stripping, the XRD and XPS of the SCMN buffer layer after cycling was performed. The XPS C 1s spectrum clearly shows that the dominant component of the SC transform into a series of lithium intercalated compounds, LiC_6_ and LiC*_x_* (x>6) after deposition. In the N 1s spectrum, the signal corresponding to Mg–N bonds have largely disappeared, replaced by characteristic peaks of Li_3_N and Li–Mg–N compounds (Figure 5d). The XRD pattern exhibits distinct diffraction peaks of newly formed phases such as Li_3_N, LiMgN, and LiC_6_, with the remaining strong signals originating from the incompletely reacted Mg_3_N_2_ precursor and the copper current collector (Figure S14). Thus, during electrochemical deposition, the reserved precursors (e.g., Mg_3_N_2_) in the top/middle layers undergo continuous lithiation, in‐situ generating highly ion‐conductive Li_3_N and lithiophilic LiC_6_ phases, exhibiting clear function‐oriented self‐optimization behavior. The dynamic *in‐situ* self‐optimization process continuously improves the ion transport and charge transfer kinetics of the interface. EIS and distribution of relaxation times (DRT) analyses were performed on symmetric cells after different cycleds at 1mAh cm^−2^ to monitor interfacial evolution (Figure S15). The bare Li symmetric cells exhibit a continuous increase in interfacial resistance, indicating progressive degradation. In contrast, the SCMN cells delivers different trend. The impedance gradually drops in the early cycles, confirming interfacial self‐optimization originating from the electrochemical conversion of residual precursors into Li_3_N and lithiophilic Li‐C/Li‐Mg alloys. A slight impedance increment appears in the subsequent long‐term cycling, yet the degradation rate is considerably lower than that of bare Li electrodes, further confirming superior interfacial stability. In the DRT results, a broad peak was observed in the range of 1E^−4^ to 1E^−2^, corresponding to *R_SEI_*. These peaks in SCMN cells show only a more modest shift, after prolonged cycling, confirming the long‐term stability of the gradient interface. The role of the SCMN interphase layer in suppressing the dendrite growth was further validated by SEM. As shown in the Figure S16, the LPSCl surface remain largely smooth and compact, with no visible dendritic structures detected after cycling in the cells with SCMN layer. In sharp contrast, the electrolyte surface in the cell without the SCMN layer exhibits distinct perforations and accumulated deposits, characteristic of lithium dendrite penetration and uneven lithium plating that has mechanically compromised the electrolyte structure [49, 50]. These results further demonstrate the superiority of the SCMN anode in achieving fast interfacial ion transport at a high current density and suppressing the growth of Li dendrites, thereby maintaining structural and electrochemical stability of the solid‐state interface.

To further evaluate the symmetric cell cycle performance under low stack pressure, the SCMN symmetric cell was assembled at 15 MPa. The Li|SCMN |LPSCl| SCMN|Li cells exhibit a high initial polarization voltage, due to the increased contact resistance under low stack pressure. As cycling proceeded, the self‐optimizing process gradually enhances interfacial contact and charge‐transfer kinetics. Consequently, the SCMN symmetric cell achieves stable cycling for over 180 h under both low stack pressure (15 MPa) and relatively high current density. The result further demonstrates that the SCMN grade buffer layer not only operates effectively under high current, but more importantly, the in‐situ self‐optimization capability enables stable interfacial performance even under limited mechanical constraints, highlighting its potential for practical applications where reduced stack pressure is desirable.

### 2.3 Electrochemical Performance of the all‐Solid‐State Cells Employing the SCMN Buffer Layer

**FIGURE 6:**
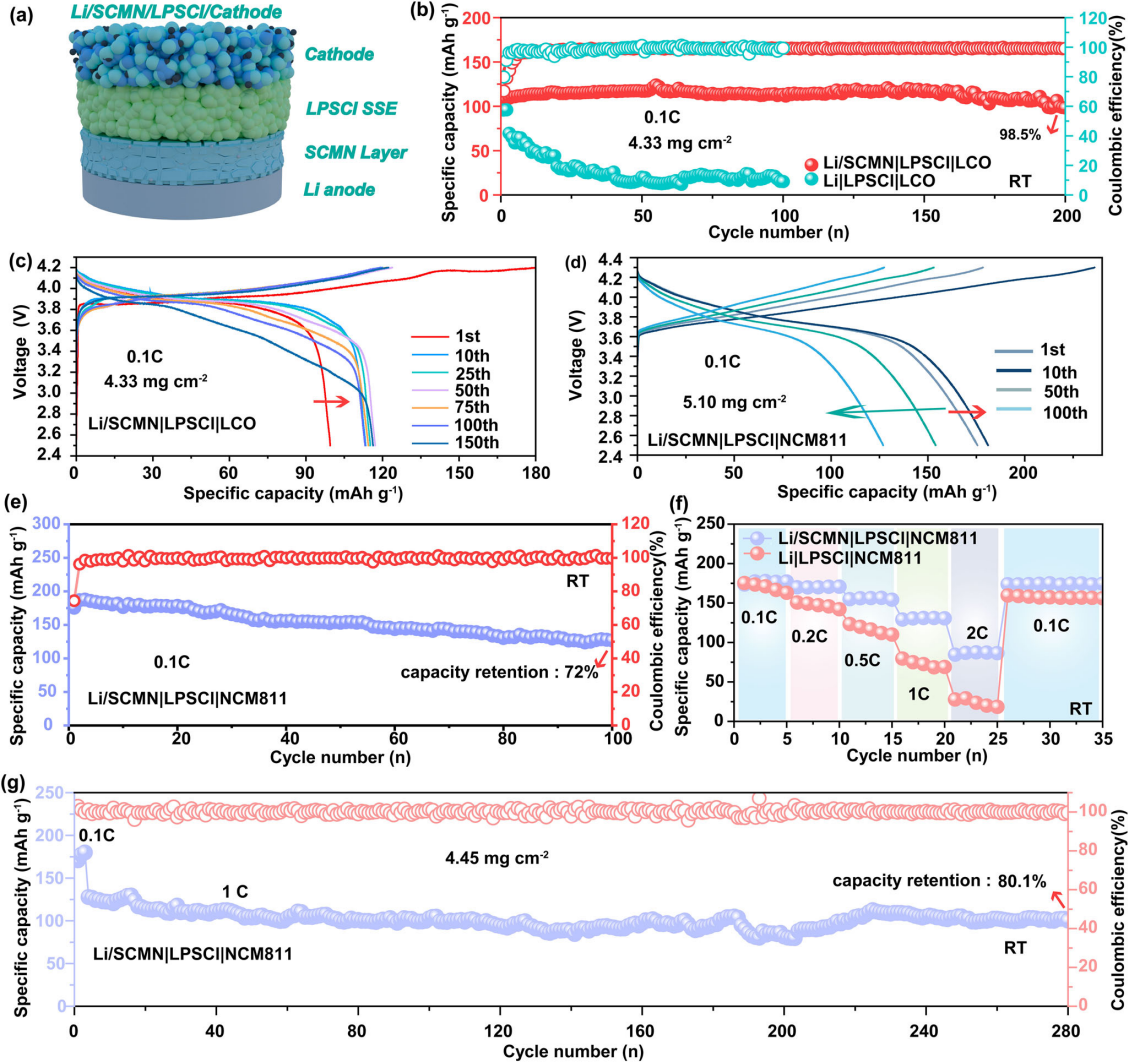
All‐solid‐state cell performances. (a) Schematic diagram of the structure of full batteries; (b) Cycle performance of LCO full cells with/without SCMN buffer layer at 0.1 C; Charge‐discharge specific capacity‐voltage curves of (c) LCO and (d) NCM811 full cells with SCMN buffer layer at 0.1 C; (e) Cycle performance at 0.1 C and (f) Rate properties of NCM811 full cells with SCMN buffer layer; (g) Cycle performance of NCM811 full cells with SCMN buffer layer at 1 C.

To evaluate the practical viability of the SCMN@Li anode, all‐solid‐state batteries were assembled with high‐voltage LCO/NCM811 cathodes (Figure 6a and Figure S17). The initial discharge capacity of the LCO full cells with SCMN layer is 99.5 mAh g^−1^ at 0.1 C with an initial coulombic efficiency (CE) of approximately 70.28%, reflecting a one‐time lithium consumption for the *in‐situ* formation of Li_3_N and LiC_x_ phases with in the SCMN interlayer. As the cycle progresses, the discharge capacity slowly increases and stabilizes at 115.2 mAh g^−1^ at the 10^th^ cycle (Figure 6c), attributable to the progressive self‐optimization of the SCMN interlayer during cycling. The dynamic evolution effectively improves the interfacial charge transport. Remarkably, the LCO full cells with SCMN layer deliver ultra‐high cycling stability, with almost no degradation after 200 cycles and a capacity retention of 98.5% (Figure 6b). In sharp contrast, the bare Li‐based full cell exhibits continuous capacity fading due to the persistent side reactions and interface deterioration between Li and LPSCl.

The NCM811 full cells with SCMN deliver an initial discharge capacity of ∼176 mAh g^−1^ at 0.1 C. Subsequently, benefiting from the in‐situ self‐optimization of SCMN, the discharge capacity slightly increases to 180 mAh g^−1^ after 10 cycles (Figure 6d). After 100 cycles, the cell presents a specific capacity of 126 mAh g^−1^ with a capacity retention of 72% (Figure 6e). On the contrary, the Li|LPSCl|NCM811 cell exhibit significant overpotential and rapid capacity degradation with low‐capacity retention of 28.2% after 100cycle (Figures S18 and S19). The EIS was employed to probe the interfacial evolution in the NCM811 full cell. As shown in the Figure S20, the Li|LPSCl|NCM811 cell exhibits a dramatically enlarged low‐frequency semicircle after cycling, indicating a substantial increase in charge‐transfer resistance due to severe interfacial degradation. Conversely, the Li|SCMN|LPSCl|NCM811 cell, despite a slightly higher initial impedance, shows reduced charge‐transfer resistance and overall internal resistance after cycling, underscoring the self‐optimization of the SCMN interlayer. Figure 6f present the rate performance of the NCNM811 full cells, where the SCMN full‐cell delivers superior discharge capacities of 177.2, 170.7, 154.2, 130.6, and 86.5 mAh g^−1^ at 0.1, 0.2, 0.5, 1.0, and 2.0 C (1 C = 200 mA g^−1^), respectively, significantly outperforming the Li|LPSCl|NCM811 cell. The remarkable reversibility is fundamentally attributed to the efficient lithium‐ion conduction and the uniform regulation of lithium deposition in the SCMN@Li anode. Furthermore, the Li|SCMN|LPSCl|NCM811 cell demonstrates outstanding high‐rate cycling stability with a capacity retention of 80.1% after 280 cycles at 1 C (Figure 6g, Figure S21). When compared with other reported sulfide‐based all‐solid‐state full cells with LCO/NCM cathodes, the capacity in this work is at an even leading level (Table S1). These results collectively affirm that the SCMN interlayer via gradient architecture and dynamic self‐optimization effectively sustains interfacial integrity and demonstrates its great potential for practical application in lithium metal batteries, offering valuable guidance for the development of future high‐energy‐density energy storage systems.

## 3 Conclusion

In summary, an adaptive gradient SC/Mg_3_N_2_ (SCMN) composite buffer layer was designed for ASSLMBs. Through a simple thermal treatment with metal Li, the SCMN layer spontaneously forms a continuous lithiophobic‐to‐lithiophilic functional gradient. After several circles of electrochemical activation, it further undergoes in‐situ phase evolution, progressively converting into highly ion‐conductive Li_3_N, highly electron‐conductive and lithiophilic Li‐C/Li‐Mg alloys. The dynamic self‐optimization process ensures sustained interfacial enhancement. The unique gradient structure, together with the formed fast ion–electron dual‐continuous network and the mechanical confinement of the soft‐carbon framework, effectively guides lithium to deposit uniformly in a bottom‐up, volume‐neutral manner within the buffer layer, thereby avoiding dendrite initiation at the solid‐electrolyte interface. As a result, the SCMN‐based symmetric cells achieve excellent long‐term cycling stability (>1600 h at 1.5 mA cm^−2^). The LCO full cells with SCMN exhibit ultra‐stable cycling stability with 98.5% capacity retention after 200 cycles. And full cell paired with a high‐voltage NCM811 cathode exhibits exceptional cycling stability (80.1%) over 280 cycles at high loading (4.45 mg cm^−2^). The reasonable interface design achieves through the space and time adaptive architecture provides a reliable approach for realizing high energy density and long lifespan of dendrite‐free all‐solid‐state lithium metal batteries.

## Author Contributions

**Yaru Shi**: conceptualization, formal analysis, software. **Libin Hu**: investigation, methodology, data curation. **Qiuhong Li**: conceptualization, investigation. **Zheng Zhang**: investigation, formal analysis. **Shoushuang Huang**: investigation, supervision, writing – original draft. **Bing Zhao**: data curation, formal analysis. **Yi Xu**: formal analysis, methodology. **Zhangjun Hu**: writing – review and editing, formal analysis, conceptualization. **Yong Jiang**: funding acquisition, investigation, data curation. **Jiujun Zhang**: conceptualization, funding acquisition, supervision, project administration.

## Conflicts of Interest

The authors declare no conflicts of interest.

## Supporting information




**Supporting File**: advs77775‐sup‐0001‐SuppMat.docx.

## Data Availability

The data that support the findings of this study are available from the corresponding author upon reasonable request.
