# Supplementary material for: A Conduction‐Nucleation‐Competition‐Guided 3D Adaptive Gradient Buffer for Dendrite‐Free All‐Solid‐State Lithium Metal Batteries
Source: Adv Sci (Weinh). 2026 Sep 27:e77775. Online ahead of print. doi: 10.1002/advs.77775 (PMC13616329; doi:10.1002/advs.77775)
Supplement: Supplementary file 1 [file ADVS-9999-e77775-s001.docx]

***Supporting Information***

**A Conduction-Nucleation-Competition‑Guided 3D Adaptive Gradient Buffer for Dendrite‑Free All‑Solid‑State Lithium Metal Batteries**

Yaru Shi^1^, Libin Hu^1^, Qiuhong Li^1^, Zheng Zhang^1^, Shoushuang Huang^1^, Bing Zhao^1^, Yi Xu^1^, Zhangjun Hu^2^, Yong Jiang^1^, Jiujun Zhang^3,4^

^1^ School of Environmental and Chemical Engineering,

Shanghai University,

Shanghai 200444, China.

Email: [jiangyong@shu.edu.cn;](mailto:jiangyong@shu.edu.cn;) [tree2000xy@shu.edu.cn](mailto:tree2000xy@shu.edu.cn).

^2^ Division of Molecular Surface Physics & Nanoscience

Department of Physics Chemistry and Biology

Linköping University

Linköping 58183, Sweden

E-mail: zhangjun.hu@liu.se

^3^ Institute for Sustainable Energy/College of Sciences,

Shanghai University,

Shanghai 200444, China.

E-mail: [jiujun.zhang@i.shu.edu.cn](mailto:jiujun.zhang@i.shu.edu.cn)

^4^ Institute for New Energy Materials and Engineering,

College of Materials Science and Engineering,

Fuzhou University,

Fuzhou 350108, China.

1. **Experimental Section**

**1.1. Material Preparation**

**Dry-Film Fabrication of the Soft Carbon/Magnesium Nitride (SCMN) Buffer Layer**

All procedures were performed in an argon-filled glove box (O₂ < 0.1 ppm, H₂O < 0.1 ppm). Soft carbon, magnesium nitride (Mg_3_N_2_), and Li_6_PS_5_Cl (LPSCI) powder were weighed at a mass ratio of 87:10:3. The mixture was transferred to a zirconia ball-milling jar with grinding beads (ball-to-powder weight ratio = 50:1) and sealed. High-energy ball milling was conducted at 300 rpm for 2 h to achieve uniform mixing. Subsequently, the composite powder was mixed with polytetrafluoroethylene (PTFE) fibers (1 wt% relative to the powder mass) in an agate mortar. The mixture was thoroughly ground and compressed to fibrillate the PTFE binder, yielding a freestanding, flexible SCMN composite film.

**Film Calendering and Integrated Anode Fabrication**

The as-prepared SCMN film was calendered using a laboratory roller press to a uniform thickness of approximately 50 μm. An integrated anode was fabricated by stacking a 50-μm-thick lithium foil and a 10-μm-thick copper foil sequentially onto the calendered SCMN film, forming a Cu|Li|SCMN sandwich structure. The multilayer stack was laminated under pressure using the roller press to ensure intimate interfacial contact. Finally, the laminated structure was thermally treated on a hot plate at 220 °C for 30 s to enhance adhesion and integration, resulting in a monolithic "integrated anode" (current collector/Li metal/SCMN buffer layer).

**1.2. Electrochemical Characterization**

**Electronic Conductivity Measurement**

The electronic conductivity of the SCMN film was measured by the direct current (DC) polarization method using a two-electrode configuration with stainless steel (SS) as blocking electrodes. A 10-mm-diameter disc of the film was placed in a zirconia sleeve cell and sandwiched between two SS rods. The assembly was pressed under 120 MPa and connected to an electrochemical workstation. A constant DC voltage of 0.1 V was applied for 1 h, and the steady-state current was recorded. The electronic conductivity ($\sigma_{e}$) was calculated using Ohm's law:

$$\sigma_{e}=\frac{I\cdot L}{V\cdot A}$$

Where *I* is the steady-state current, *L* is the film thickness, *V* is the applied voltage, and *A* is the electrode area.

**Ionic Conductivity Measurement via Electron-Blocking Cell**

The ionic conductivity of the SCMN film was evaluated using a symmetric Li|SCMN|Li configuration with Li_6_PS_5_Cl as the ionically conductive separator and electron-blocking layer. A 10-mm-diameter SCMN disc was sandwiched between two LPSCI pellets (60 mg each) inside a zirconia sleeve cell. Two stainless steel plungers were used to apply a constant pressure of 360 MPa. Electrochemical impedance spectroscopy (EIS) was performed from 1 MHz to 0.1 Hz with an AC amplitude of 10 mV. The ionic conductivity ($\sigma_{i}$) was derived from the high-frequency intercept of the Nyquist plot using the formula:

$$\sigma_{i}=\frac{L}{R_{b}\cdot A}$$

where$R_{b}$ is the bulk resistance obtained from the EIS fitting.

**Symmetrical Cell Assembly and Tests**

The entire cell assembly was performed in an argon-filled glove box (H₂O < 0.1 ppm, O₂ < 0.1 ppm). A solid electrolyte layer was fabricated by compressing 120 mg of LPSCl powder in a PEEK model at 375 MPa for 5 minutes using a hydraulic press, resulting in a dense and flat pellet. Subsequently, the ejector pins were removed, and integrated anodes were symmetrically placed against the pellet. The stack was pressed again at 100 MPa for 1 minute and finally secured with bolts to form a symmetrical solid-state battery. Galvanostatic lithium plating/stripping and critical current density (CCD) tests were carried out at room temperature at various current densities using a LAND-CT 2001A system. Electrochemical impedance spectroscopy (EIS) was performed with a Solartron Analytical 1400 system from 1.0 MHz to 0.01 Hz with an amplitude of 5 mV at room temperature.

**Full Cell Assembly and Tests**

The full cells assembling process were conducted in an argon-filled glove box (H_2_O < 0.1 ppm, O_2_ < 0.1 ppm). The composite cathode was prepared by thoroughly grinding LCO/NCM811, LPSCl, vapor-grown carbon fiber (VGCF), and Super P at a mass ratio of 7:2:0.5:0.5 for 30 minutes. An all-solid-state lithium metal battery was assembled by first pressing 120 mg of LPSCl SE powder in a 10 mm diameter mold at 375 MPa for 5 min to form a dense electrolyte pellet. Then, the cathode composite was uniformly dispersed onto one side of the pellet and pressed at 375 MPa for 5 min. The anode (integrated anode/Li anode) was applied on the opposite side and compressed at 225 MPa for 2 min. The cell was finally fixed with mold bolts for testing.

Galvanostatic charge–discharge tests were conducted using a LAND-CT 2001A system within a voltage range of 2.5–4.2 V (vs. Li/Li⁺) at various C-rates. The electrochemical performance was evaluated in terms of cycling stability, Coulombic efficiency, and capacity retention. Electrochemical impedance spectroscopy (EIS) was measured with a Solartron Analytical 1400 system from 1.0 MHz to 0.01 Hz using a 5mV amplitude at room temperature.

**Figure S1:** (a) Schematic diagram of the buffer layer; Surface optical images of (b) Top layer, (b) Middle layer and (c) Bottom layer (Alloy layer).

**Figure S2:** SEM image of “peeling” top film from the middle layer

**Figure S3:** XPS spectra of the (a) top layer, (b) middle layer and (c) bottom layer in the buffer layer.

**Figure S4:** (a) Ternary phase diagram of Li-Mg-N ternary compounds; (b) Schematic composition diagram of the stratified structure of SCMN.

**Figure S5:** Raman spectra of (a) Mg_3_N_2_ and (b) SC.

**Figure S6:** Raman spectra of the (a) top layer, (b) middle layer and (c) bottom layer in the SCMN buffer layer.

**Figure S7:** Adsorption structure models of lithium atoms on (a) Li (100) and (b) Mg (001) surfaces and the corresponding binding energy.

**Figure S8:** Model of lithium-ion intercalation reaction into carbon and reaction Gibbs free energy.

By calculating the Gibbs free energy of lithium-ion intercalation into carbon using DFT, it was found that ΔG = -2.29 eV (spontaneous reaction), which is lower than the lithium adsorption energy on the graphite surface, confirming that lithium insertion into the soft carbon substrate takes precedence over surface nucleation. This phenomenon not only inhibits lithium deposition within the buffer layer and at the interface, but also weakens the lithiophilic nature of the interlayer by forming a lithium-carbon alloy.

**Figure S9:** Lithium-ion migration barrier for LiC_6_ (001) and SC (001).

**Figure S10:** DC resistance and electron conductivity of each layer and soft carbon

**Figure S11:** Capacity - voltage curve of lithium unidirectional deposition.

**Figure S12:** Solid electrolyte surface after lithium deposition with (a) and without (b) SCMN membrane.

**Figure S13:** (a) SCMN membrane surface before deposition; SCMN membrane surface at a deposition capacity of (b) 1.5 mAh cm^-2^ and (c) 3 mAh cm^-2^.

**Figure S14:** XRD pattern of the SCMN membrane after lithium deposition.

**Figure S15:** EIS and DRT characterization of interfacial evolution in (a) SCMN and (b) bare Li symmetrical cells.

**Figure S16:** Top-view SEM of the LPSCl SE surfaces after cycling in the lithium symmetric cells with/without SCMN layer.

**Figure S17:** Top-view of LCO cathode particles.

As shown in the **Figure S17**, the cathode particles remain intact without trans granular fracture or pulverization and the VGCF fibers retain their high-aspect-ratio morphology, forming a continuous conductive network throughout the composite cathode.

**Figure S18:** Cycle performance of the NCM811 full cell without SCMN buffer layer at 0.1 C.

**Figure S19:** Charge-discharge specific capacity-voltage curves of NCM811 full cells without SCMN buffer layer at 0.1 C.

**Figure S20:** EIS of NCM811 full cells with/without SCMN buffer layer before and after cycling.

**Figure S21:** Charge-discharge specific capacity-voltage curves of NCM811 full cells without SCMN buffer layer at 1 C.

**Table S1:** Electrochemical comparison with the previously published sulfide solid-state lithium-metal cells.

| Cells | Specific Capicity  （mAh g^-1^） | Ref. |
| --- | --- | --- |
| LCO/LPSC-MF/Li | 112.9 (0.1C) | *Angew. Chem. Int. Ed.* 2023, 62, e202302655 |
| LCO/LPS/LGC-Li | 116.4 (0.1C) | *Energy Storage Mater. 2023, 63,103009* |
| Li\| Li_6.12_P_0.94_In_0.06_S_4.91_O_0.09_Cl\|LCO | 115.5 (0.1C) | *Chem. Engin. J. 2024, 498, 155538* |
| NCM/LPSCl_1.5_/Li-In | 155.4 (0.2C) | *Adv. Funct. Mater.* 2022, 32, 2207978 |
| NCM@LPSCl/NCM/Li | 164.1 (0.1C) | Adv. Funct. Mater. 2023, 33, 2211355 |
| NCM/Li_5.5_PS_4.5_Cl/Li | 165 (0.2C) | *Angew. Chem. Int. Ed.* 2023, 135, e202213228 |
| LNO@NCM/Li_6.04_P_0.98_Bi_0.02_ S_4.97_O_0.03_Cl/Li | 131.6 (0.1C) | *Adv. Funct. Mater.* 2022, 2203858 |
| NCM523@LiNbO_3_/Li_3.12_P_0.94_ Bi_0.06_S_3.91_O_0.09_ | 130 (0.1C) | *Adv. Funct. Mater.* 2022, 2205998 |
| Li/SCMN\|LPSCl\|LCO | 115.2 (0.1C) | This work |
| Li/SCMN\|LPSCl\|NCM811 | 177.2 (0.1C)  170.2 (0.2C) |  |
